# CONNECTING COMMUNITIES: ROLES OF AGING ATTITUDES, PLACE ATTACHMENT, AND PSYCHOLOGICAL WELL-BEING

**DOI:** 10.1093/geroni/igad104.1180

**Published:** 2023-12-21

**Authors:** Su-I Hou, Li-Fan Liu

## Abstract

As the global population ages, communities must find innovative ways to support older adults. This symposium focuses on the intersections of aging, community, and culture, particularly emphasizing the importance of place attachment, aging attitudes, psychological well-being, and engagement in promoting healthy aging. Leading experts from social gerontology, psychology, and public health across the globe will explore the complex relationships between aging, community, and culture. Drs. HM Chen from Texas A&M University–Central Texas (TAMCT) and Hou from the University of Central Florida (UCF) from the United States will start the symposium with a systematic literature review on culture and aging in the community among Chinese older adults. Drs. JJ Chen and Liu from the National Cheng-Kung University (NCKU) will examine the mediating effect of psychological place attachment, how older adults perceive their living environments, and (positive versus negative) attitudes towards aging and psychological well-being. Dr. Hou from UCF will further assess aging attitudes (engagement versus enjoyment) among older adults participating in neighborhood lunch versus lifelong learning programs to discuss the implications of tailored aging service development. Finally, Drs. Lin and Fung from The Chinese University of Hong Kong (CUHK) will discuss the association between activity diversity and psychological well-being among community-dwelling older adults in Hong Kong. Through interdisciplinary dialogue and sharing of lessons learned, this global symposium promotes a deeper understanding of the roles of aging attitudes, place attachment, culture, and psychological well-being among older adults to foster innovative approaches to promoting healthy aging and community engagement. This is an Aging Among Asians Interest Group Sponsored Symposium. This is a collaborative symposium between the Aging Among Asians and International Comparisons of Healthy Aging Interest Groups.

